# Multivariate Statistical Techniques: A New Approach to Identify the Commercial Properties of Mixtures of Flours of *Lentinula edodes* and Cocoa Pod Husk

**DOI:** 10.3390/jof9100991

**Published:** 2023-10-06

**Authors:** Juan Diego Valenzuela-Cobos, Fabricio Guevara-Viejó, Ana Grijalva-Endara, Purificación Vicente-Galindo, Purificación Galindo-Villardón

**Affiliations:** 1 Centro de Estudios Estadísticos, Universidad Estatal de Milagro (UNEMI), Milagro 091050, Ecuador; juan_diegova@hotmail.com (J.D.V.-C.); jguevarav@unemi.edu.ec (F.G.-V.); purivg@usal.es (P.V.-G.); 2 Facultad de Ciencias Químicas, Universidad de Guayaquil, Guayaquil 090514, Ecuador; anagrijalvae@gmail.com; 3 Department of Statistics, University of Salamanca, 37008 Salamanca, Spain; 4 Institute for Biomedical Research of Salamanca (IBSAL), 37007 Salamanca, Spain; 5 Centro de Investigación Institucional, Universidad Bernardo O’Higgins, Av. Viel 1497, Santiago 8370993, Chile

**Keywords:** cocoa pod husk, *Lentinula edodes*, multivariate statistical techniques, chemical and commercial properties

## Abstract

*Lentinula edodes* is known to show high nutritional and organoleptic properties and can be mixed with different by-products in the production of new foods with important functional characteristics. Cocoa pod husk (CPH) is the main by-product in the cocoa industry and presents important bioactivities. In this research, two mixtures were applied based on *Lentinula edodes* mushroom flour from fifty different strains (strain 1 to strain 50) and cocoa pod husk flour (CPHF): 60% *Lentinula edodes* mushroom flour from each strain mixed with 40% CPHF (Mixture 1), and 80% *Lentinula edodes* mushroom flour from each strain mixed with 20% CPHF (Mixture 2). The parameters evaluated were moisture, proteins, fat, total dietary fiber (TDF), ash, carbohydrates, antioxidant capacity, antimicrobial activity, pH and water activity (a_w_). The multivariate statistical techniques evaluated those samples that exhibited the highest degree of correlation with the distinct chemical and commercial parameters, showing that sample 13 of both mixture flours (M1 and M2) obtained by the mushrooms of *L. edodes* (strain 13) with CPH indicated the higher significant of the parameters. In addition, the sensory test of the best flour (sample 13) was applied to 50 panelists, presenting the best sensory characteristics, such as color, aroma, texture and general acceptability.

## 1. Introduction

*Lentinula edodes* (shiitake) is the mushroom with the second-highest production worldwide, and represents about 22% of the total production of edible mushrooms [1]. The traditional method of cultivating *Lentinula edodes* is in logs cut from trees and requires long periods of time, causing negative impacts on the environment due to the forest destruction of the trees. The high consumption rates of this edible fungi have promoted the development of technologies based on the use of agricultural waste with high carbon and nitrogen content as substrates in the cultivation of *Lentinula edodes*. The nutritional, pharmaceutical and organoleptic properties of *Lentinula edodes* are related to the nutrient composition of the culture substrate and the type of supplements added [2,3]. Furthermore, the fresh shiitake mushroom presents aromatic substances, improving sensory characteristics such as flavor, allowing products processed through different techniques such as drying, enzymatic hydrolysis and fermentation to be obtained [4]. Studies have shown the importance of using the *Lentinula edodes* mushrooms to enrich the nutritional value of food products because they are appetizing and nutritious. *Lentinula edodes* protein is composed of all essential amino acids in ideal proportions for human nutrition. Additionally, *Lentinula edodes* shows a high composition of complex carbohydrates, vitamins and minerals and a high amount of trace elements that improve physical fitness and prevent diseases [5]. Consequently, *Lentinula edodes* mushrooms can be mixed with various types of products or by-products such as cocoa waste to obtain complementary foods with high nutritional content.

Cocoa production worldwide represents around 4.7 million tons of cocoa beans, and is found mainly in African and American countries. There are four different cocoa varieties: National, Criollo, Forastero and Trinitario. Forastero is the variety with the highest harvest in Africa and has important characteristics such as a greater resistance to different pests and higher productivity in comparison to other varieties [6]. Ecuador is the country with the highest production of cocoa of the “National” variety, which is characterized by a floral, blackcurrant and spicy smell [7,8]. Ecuador generates tons of waste that come from the cocoa industry; the main by-product is the cocoa pod husk, which represents 75% of the total weight of the fruit [9,10]. For each kilogram of cocoa beans, 10 kg of CPH is produced, which is generally used as a fertilizer, in soap production, as animal feed and in the production of absorbents for different contaminants such as heavy metals, dyes, pharmaceuticals and pesticides [11,12]. The CPH in Ecuador is generally used as an organic material for different crops; nonetheless, this process generates environmental problems such as soil contamination produced by the generation of leachates with high concentrations of ammoniacal nitrogen due to the slow biodegradation of this by-product [13,14]. The CPH shows the highest content of protein, ranging from 7 to 10%, ash content of 6.4 to 8.4%, total dietary fiber between 36.6 and 56.1%, carbohydrates from 32 to 47% and moisture ranging from 85 to 90.5%, and also presents a low content of fat, between 1.5 and 2% [15,16,17]. In the agricultural sector, the effective visualization of data, relations and trends pertaining to chemical and nutritional parameters has been facilitated through the application of multivariate statistical analysis and data mining techniques, perceiving unnoticed relationships compared to the traditional methods [18,19].

Multivariate statistical techniques allow the study, representation and interpretation of data resulting from the observation of more than two statistical variables on a sample of individuals; these variables are homogeneous and correlated [20]. Multivariate methods and data mining techniques allow complex problems that have different variables and levels of factors to be solved, and among the main statistical algorithms we have the PCA Biplot and GGE Biplot, with their respective cluster analysis. 

Biplots allow the study of sets of variables in a population of individuals and the determination of data sets with the same characteristics through the use of mathematical operations derived from geometry, matrix calculus and multivariate distributions [21]. These multivariate methods allow the extraction of new principal components that have greater variance compared to the original components. The new variables are linear combinations that are built according to the total variability that they collect from the sample. Principal component methods do not require data to be normal; however, data showing normal distribution may give a better interpretation of principal components [22].

The main purpose of this research was to use statistical algorithms, GGE Biplot and PCA biplot, to determine the flour mixtures (formulated from different proportions of *Lentinula edodes* mushroom flour and the cocoa pod husk flour) that present the highest chemical and commercial characteristics, such as moisture, proteins, fats, total dietary fiber (TDF), ashes, carbohydrates, antioxidant capacity, antimicrobial activity, pH and water activity (a_w_). Additionally, the flour mixtures with the highest chemical and commercial characteristics were subjected to a sensory test that was evaluated using the ANOVA test.

## 2. Materials and Methods

### 2.1. Collection of the Fungal Strain

The strains of *Lentinula edodes* were obtained from the fungal collection of the Research and Development Laboratory of Ecuahidrolizados. A total of fifty distinct strains were employed and subsequently cultured on PDA plates at a temperature of 25 °C. The strains were labeled as strain 1 to strain 50.

### 2.2. Production of the Fruit Bodies

The fruiting bodies of the 50 different strains of *Lentinula edodes* were obtained using a mixture of agricultural waste such as oak sawdust (44%), banana leaves (30%), millet seed (16%), cotton seed husk (5%) and CaCO_3_ (5%). The fruiting conditions were as follows: relative humidity between 85 and 90%, temperature of 25 ± 1 °C, air recirculation and a lighting period of 12 h [23]. The cultures for each *Lentinula edodes* strain were performed in triplicate.

### 2.3. Obtention of the Flours

CPH are among the main by-products of the Ecuadorian food industry, and is generated by the high production of fine chocolate. Therefore, it was collected from the AgroFarm Sánchez-Jaramillo in the Province of Manabí. The CPH were placed in boiling water for 10 min to inactivate the enzyme. Then, the fruit bodies of the fifty mushroom strains and the CPH were exposed to direct sunlight until they were dry.

The mushrooms and the CPH were ground to powder in a paddle mill for 30 s and passed through a sieve of 200 µm mesh, obtaining fungi flour and cocoa pod husk flour (CPHF), respectively, and the flours were stored at 4 °C.

### 2.4. Composition of the Mixtures

The mixtures were composed using the following formulations:

Mixture 1—60% mushroom flour from each *Lentinula edodes* strain mixed with 40% CPHF. Fifty samples (1–50) were obtained for the different strains collected.

Mixture 2—80% mushroom flour from a specific *Lentinula edodes* strain mixed with 20% CPHF. Fifty samples (1–50) were obtained for each of the different strains collected.

### 2.5. Chemical Composition

The chemical parameters of the mixtures were analyzed according the AOAC procedures [24] with certain modifications: moisture content analyzed via the oven method, proteins (Nx6.25) calculated using the Kjeldahl method, fat measured by extracting the weight of sample with hexane using a Soxhlet apparatus, total dietary fiber (TDF) using sulfuric acid H_2_SO_4_ (1.25%) and sodium hydroxide NaOH (1.25%) (enzymatic-gravimetric method), ash from the calcination of the sample at 550 °C and carbohydrates determined by using Equation (1):

C (%) = 100 − (%moisture + %protein + %fat + %TDF + %ash contents)(1)

Equation (1). Percentage of carbohydrates of mixtures.

### 2.6. Commercial Properties

The calculated commercial properties of the flour mixtures were antioxidant capacity, antimicrobial activity and technological properties such as pH and water activity (a_w_).

#### 2.6.1. Antioxidant Capacity

The antioxidant capacity was determined using the extracts of the flour mixtures. They were prepared by dissolving 2 g of each flour mixture in 40 mL of methanol at 100 rpm, which was maintained at 28 °C for a duration of 2 h. Subsequently, the solution was filtered, followed by dissolution in another aliquot of methanol. The two methanol fractions were subjected to evaporation using a rotary evaporator, resulting in a concentrated liquid fraction ultimately achieving a concentration of 30 mg/mL.

The extracts of the flour mixtures were analyzed to determine the antioxidant capacity, and 30 μL of the extract and 270 μL of DPPH methanolic solution (6 × 10^−5^ mol L^−1^) were mixed in a 96-well plate. The mixture of the extract with ethanol was incubated in the dark for 30 min and later it was measured at 515 nm using a microplate reader. The radical scavenging activity (RSA) of DPPH was determined using Equation (2) [25].

(2) $$RSA(\%)=\frac{ADPPH-AS}{ADPPH}\times 100$$

Equation (2). DPPH radical scavenging activity (RSA).

#### 2.6.2. Antimicrobial Activity

The antimicrobial activity was determined using the following Gram-positive bacteria: *Salmonella typhimurium* (ABN 572). The bacteria have been deposited at Research and Development Laboratory of Ecuahidrolizados.

A saline solution with a concentration of 1.0 × 10^6^ CFU/mL was used to adjust the bacterial suspensions. The extracts of the flour mixtures were dissolved in 30 mL of ethanol, and the ethanolic extracts were evaporated using a rotary evaporator. The extracts were dissolved in 5% DMSO (1 mg mL^−1^) and added into a Tryptic Soy Broth (TSB) medium (100 μL) with bacterial inoculum (1.0 × 10^5^ CFU per well) at a final volume of 100 µL.

The microplates were subjected to incubation at 170 rpm for one day at a temperature of 38 °C. Subsequent to this incubation period, a solution of commercial dye, hexamethylpararosaniline chloride, commonly known as crystal violet, was introduced at a concentration of 0.1 mg/mL. A further incubation of thirty minutes followed this addition. Samples that demonstrated growth inhibition exhibited a distinct color change. The determination of the bacterial growth inhibitory concentrations (MIC) was accomplished through the microscopic examination of a subculture of the bacterial extract in microtitre plates, which were subsequently incubated for a period of 24 h. The optical density of each well was measured and compared to both a blank and a positive control, the latter being represented by streptomycin [20]. 

#### 2.6.3. Technological Properties

The pH levels of the mixtures were measured with a pH 400 m (Spectrum Technologies Inc., Aurora, IL, USA), diluting the mixtures in distilled water (1:10), and the water activity (a_w_) for mixtures was measured with a Labmaster AW (Novasina AG, Lachen, Switzerland) at 25 °C [26].

### 2.7. Sensory Analysis Test

The sensory evaluation test was carried out by 50 panelists during a period of 1 h. The panelists received educational training for 5 min before participating in the sensory test, and the samples of food were provided in a random order. The 4 samples evaluated were only mushroom flour of *L. edodes* strain 13, only CPH flour, mixture 1 (60% mushroom flour from the *L. edodes* strain 13 and with 40% CPH flour) and mixture 2 (80% mushroom flour from the *L. edodes* strain 13 and with 20% CPH flour). The survey presented 9 points: 1 = disliked extremely, 2 = disliked very much, 3 = disliked moderately, 4 = disliked slightly, 5 = neither liked nor disliked, 6 = liked slightly, 7 = liked moderately, 8 = liked very much, 9 = liked extremely. The sensory parameters evaluated were color, aroma, texture and general acceptability [27,28].

### 2.8. Statistical Analysis

The chemical composition and commercial characteristics (moisture, proteins, fat, total dietary fiber (TDF), ash, carbohydrates, antioxidant capacity, antimicrobial activity, pH and water activity (a_w_)) were measured in triplicate, and the data were subjected to multivariate statistical techniques such as GGE Biplot and PCA Biplot using R software v.4.1.1.

In addition, the results obtained from the sensory analysis survey were tabulated and subsequently examined using one-way analysis of variance (ANOVA) at the *p* < 0.05 level to determine significant differences between sensory parameters such as color, aroma, texture and acceptability. If statistical differences were found, Duncan’s test was applied with α = 0.05. Analyzes were carried out using statistical software (Statgraphic v.16).

#### 2.8.1. GGE Biplot

A GGE biplot graph is used to obtain the visualization of the best behavior of a certain genotype in each environment. This is undertaken through interconnecting all the genotypes that are at the extreme points of the graph and their respective perpendicular lines, forming a polygon. This polygon shows the best genotype for each environment and divides the environments into different groups.

In the event that the genotypes adapt to different environmental groups and the variation between groups is greater than within the group, a mega-environment is formed, which indicates the genotypes that have a broad or specific adaptation to certain environments or groups of environments.

The GGE Biplot model keeps G and GE together and partitions GGE into two multiplicative terms, and therefore the equation is given by [29,30]:

$$Y_{ij}=\mu +e_{j}+\lambda_{1}\gamma_{i1}\alpha_{j1}+\lambda_{2}\gamma_{i2}\alpha_{j2}$$

where:

$Y_{ij}=$ The performance of genotype i in environment j;
μ= Global average;
$e_{j}=$ Effect of the environment j;
$\lambda_{1}=$ Eigenvalue of the first principal component;
$\lambda_{2}=$ Eigenvalue of the second principal component;
$\gamma_{i1}=$ Eigenvectors of the genotype i associated with the first component;
$\gamma_{i2}=$ Eigenvectors of the genotype i associated with the second component;
$\alpha_{j1}=$ Environmental eigenvectors j for the first component;
$\alpha_{j2}=$ Environmental eigenvectors j for the second component;
$\varepsilon_{ij}=$ Experimental error.

#### 2.8.2. PCA Biplot

The PCA biplot allows a standardized data set to be obtained in order to extract the main patterns of the potential groups and explore the correlations between the objective indicators. The PCA biplot decreases the amount of the data and shows the relative position of each indicator in a quadrant of the axes of the plane.

The PCA biplot method approximates the matrix X by $U_{\left(q\right)nxq}$, with an adequate factorization, consequently obtaining the expression [31]:

$$X≅X_{\left(q\right)}≅U\sum VT≅E_{\left(q\right)}G_{\left(q\right)}^{T}$$

where $E_{\left(q\right)}$ and $G_{\left(q\right)}$ are full rank matrices, defined as:

$$E_{\left(q\right)}=U_{\left(q\right)}\sum_{\left(q\right)}^{p}$$

and

$$G_{\left(q\right)}=V_{\left(q\right)}\sum_{\left(q\right)}^{1-p}$$

depending on the value selected for p(0≤p≤1).

## 3. Results and Discussion

The research purpose was to identify the flour mixtures with the highest chemical characteristics and commercial properties of *Lentinula edodes* flour and CPHF.

The samples of the flour mixtures were made according to the following distribution:

1–50: Flour samples corresponding to Mixture 1 (60% mushroom flour from a specific *Lentinula edodes* strain mixed with 40% CPHF) or flour samples corresponding to Mixture 2 (80% mushroom flour from a specific *Lentinula edodes* strain mixed with 20% CPHF).

### 3.1. Statistical Algorithm for Chemical Characteristics of the Mixtures

**Figure 1:**
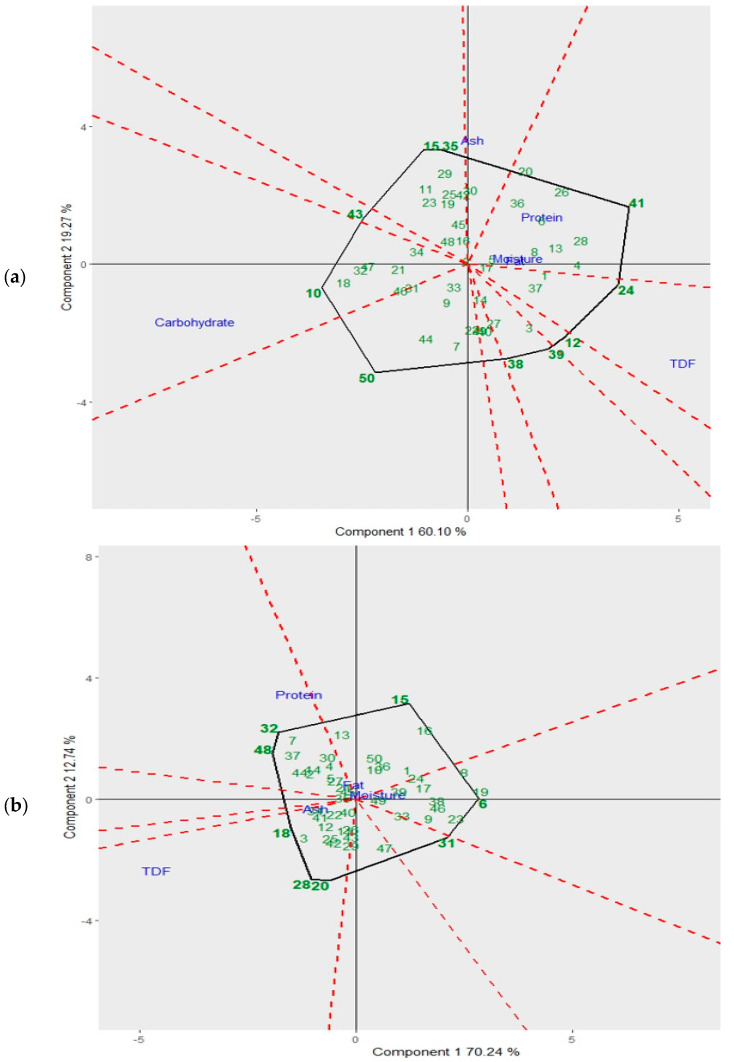
GGE biplot for chemical characteristics of the flour mixtures samples. (**a**) GGE biplot of the samples of the flour mixture M1 (sample 1 to sample 50); (**b**) GGE biplot of the samples of the flour mixture M2 (sample 1 to sample 50).

The GGE biplots present the chemical composition of the samples corresponding to mixtures 1 and 2. Figure 1a presents the GGE biplot with a cumulative inertia of 79.37%, while Figure 1b shows the GGE biplot with a cumulative inertia of 82.98%. The clusters were formed using six variables such as moisture, protein, fat, total dietary fiber (TDF), ash and carbohydrates. The inertia percentage uses criteria to choose the most significant flour samples on the screen graph.

Figure 1a indicates that eight samples of the flours of mixture 1 (5, 6, 8, 13, 20, 26, 28, 36, 41) presented the highest content of protein, moisture and fat; four samples of the flours of mixture 1 (1, 17, 24, 37) showed the highest content of total dietary fiber (TDF); eight samples of mixture 1 (10, 18, 21, 31, 32, 34, 46, 47) presented the highest values of carbohydrates; and one sample of mixture 1 (30) indicated highest value of ash. On the other hand, Figure 1b points out that 13 samples of the flours of mixture 2 (2, 4, 5, 7, 13, 14, 27, 30, 32, 37, 44, 45, 48) presented the highest content of protein, moisture and fat, and 13 samples of the flours of mixture 2 (3, 12, 18, 20, 22, 25, 26, 28, 29, 40, 41, 42, 43) showed the highest values of ash and total dietary fiber (TDF). Sample 13 of both mixtures’ flours (M1 and M2) obtained using the mushrooms of *L. edodes* (strain 13) with CPH presented the highest values in three of the six variables of the analysis: protein, moisture and fat.

In the following study, evaluating the potential of CPH as a food additive, it was found that this by-product is rich in total dietary fiber (35.3/100 g dry weight of the husk), carbohydrates (49.3/100 g) and ash (8.9/100 g) [27]. Other research has shown that the CPH is a fibrous material due to its composition in cellulose, hemicellulose, lignin and pectin [9]. Ribeiro et al. [32] indicated the nutritional composition of the *Lentinula edodes* mushroom, showing the highest values in moisture (90/100 g), total dietary fiber (4.6/100 g) and protein (1.6/100 g) and important levels in amino acids, polysaccharides, vitamins and minerals [33]. 

The use of two different ingredients in the mixtures, such as *Lentinula edodes* mushroom flour and the CPHF, that present high protein and fiber values allows new food products with an important nutritional composition to be obtained, promoting the cultivation of the *Lentinula edodes* mushrooms and also the use of a by-product such as CPH in the food industry.

### 3.2. Statistical Algorithm for Commercial Properties of the Mixtures

**Figure 2:**
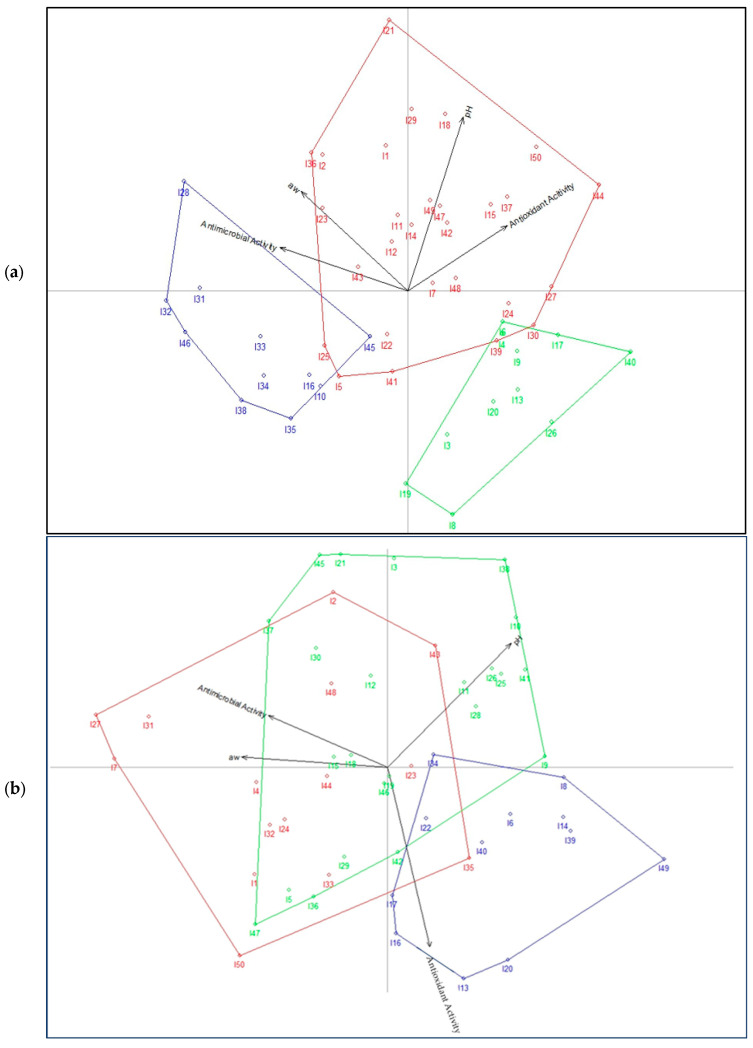
PCA biplots for commercial properties of the flour mixtures samples. (**a**) PCA biplot of the samples of the flour mixture M1 (sample 1 to sample 50); (**b**) PCA biplot of the samples of the flour mixture M2 (sample 1 to sample 50).

Figure 2 presents the formation of three groups using four variables such as antioxidant capacity, antimicrobial activity, pH and water activity (a_w_). Figure 2a indicates the PCA biplot with a cumulative inertia of 57.7%, while Figure 2b shows the PCA biplot with a cumulative inertia of 55%. Inertia allows a quick and accurate evaluation of the most important points (flour samples) of the factorial plane.

Figure 2a indicates the presence of three important groups of samples in mixture 1: Cluster 1, “color blue”, formed by 11 samples (10, 16, 28, 31, 32, 33, 34, 35, 38, 45, 46), that indicated the highest relation with the antimicrobial activity; Cluster 2, “color red”, formed from 28 samples (1, 2, 5, 7, 11, 12, 14, 15, 18, 21, 22, 23, 24, 25, 27, 29, 30, 36, 37, 39, 41, 42, 43, 44, 47, 48, 49, 50), which presented the highest values of antioxidant activity, pH and water activity (a_w_); and Cluster 3, “color green”, formed of 11 samples (3, 4, 6, 8, 9, 13, 17, 19, 20, 26, 40), which showed highest values of antioxidant activity and pH. On the other hand, Figure 2b shows the presence of three big groups of samples of mixture 2: Cluster 1, “color red”, was formed of 15 samples (1, 2, 4, 7, 23, 24, 27, 31, 32, 33, 35, 43, 44, 48, 50) that indicated the highest relation with the water activity (a_w_); Cluster 2, “color green”, was formed of 22 samples (3, 5, 10, 11, 12, 13, 18, 19, 21, 25, 26, 28, 29, 30, 36, 37, 38, 41, 42, 45, 46, 47) and presented the highest values of antimicrobial activity and pH; and Cluster 3, “color blue”, was formed of 12 samples (6, 8, 13, 14, 16, 17, 20, 22, 34, 39, 40, 49) and presented the highest values of antioxidant activity. Sample 13 of both mixtures’ flours (M1 and M2) obtained using the mushrooms of *L. edodes* (strain 13) with CPH presented the highest values of antioxidant activity and pH. Sample 13 also showed the highest values of antimicrobial activity in the mixture M2, establishing itself as the strain with the best characteristics. 

*Lentinula edodes* is an edible mushroom that contains polyphenols and polysaccharides that indicate a high antioxidant capacity [34]. Antioxidants prevent the formation of free radicals and other compounds that damage DNA, lipids, proteins and other biomolecules [35]. In addition, studies have shown similar results regarding the effectiveness of the antimicrobial activity of aqueous extracts of *Lentinula edodes* against Gram-positive bacteria. The antimicrobial capacity is related to the total content of phenols and ortho-diphenols that are produced in the different extracts (aqueous or methanol), and also the phenolic compounds depend on the morphological and genetic characteristics of the fungi strain [36]. At present, *Lentinula edodes* is known for its importance in the pharmaceutical area, especially in the prevention of hypercholesterolemia and diabetes [37]. In addition, the CPHF has presented compounds such as PCA (3,4-dihydroxy benzoic acid), epicatechin and procyanidins that are related to antioxidant capacity [38]. Different research works have shown the effectiveness of methanolic extracts from CPH against Gram-positive bacteria such as *S. aureus* and Gram-negative bacteria such as *E. coli*, obtaining higher values in soluble methanol in comparison with soluble ethyl acetate [39]. The inhibition capacity of CPH against bacteria is influenced by the phenolic components that act on the cell wall of the bacteria, causing the death of the microorganism. In addition, the inhibition effect is related to the hydrophilic compounds that surround the outer membrane proteins of the Gram-negative bacteria [40]. The pH value is a variable that influences the obtaining of pectin and therefore the physicochemical properties of food. Pectin allows the texture and stability in food products to be improved [41].

GGE and PCA biplots indicated that flour samples of mixtures 1 and 2 that were obtained from the fruit bodies of *Lentinula edodes* strain 13 mixed with CPH showed important values of nutritional composition, antimicrobial and antioxidant activities and pH that are characteristic for formulating new products and also can be used in the pharmaceutical sector.

### 3.3. Sensory Characteristics of the Mixtures

*Lentinula edodes* mushrooms present volatile compounds, alcohols, aldehydes, ketones, acids, esters, sulfur compounds and pyrazines, that contribute to the quality and flavor of new foods [42]. Volatile compounds are produced in food processing by chemical reactions such as lipid oxidation and degradation, the Maillard reaction or caramelization reactions [43]. Dried *Lentinula edodes* mushrooms have a toasted aroma or garlic aroma, so they can be used for the development and research of products in the food industry. Currently, the fruiting bodies of mushrooms are evaluated in the condiment industry for their flavor. The high content of compounds of eight carbons, sulfur and aldehydes that present in the composition of the fruiting bodies of *L. edodes* come from the sawdust substrate that is used in their cultivation [44]. Results in sensory tests indicated the highest odor and chewiness preferences among the panelists when the food products had a greater amount of *L. edodes* mushrooms in their composition [45]. Cocoa pod husk flours present volatile aromatic compounds when subjected to thermal processes due to the formation of substances such as methylpyrazines and heterocyclic nitrogen responsible for the sweet and strong aroma, xanthines and theobromine related to caffeine and bitter flavors, respectively. In another research work, consumers have reported the general acceptability of the dark color of CPH in the production of new foods [46]. The CPH has presented characteristics as functional additives in meat products such as pork sausages [47].

The results obtained by the 50 panelists who carried out the sensory evaluation indicated that the sample of only mushrooms flour of *L. edodes* strain 13, in mixture 2 (80% mushroom flour from the *L. edodes* strain 13 and with 20% CPH flour) and mixture 1 (60% mushroom flour from the *L. edodes* strain 13 and with 40% CPH flour), presented the highest values of aroma, while mixture 2 and the sample of only mushroom flour of *L. edodes* strain 13 showed color values and mixture 2 indicated the highest values of texture and acceptability. The panelists indicated that mixture 2 presented the highest sensory attribute values compared to the other samples (Table 1).

## 4. Conclusions

The GGE and PCA biplots showed that the flour samples of the mixtures 1 and 2 that were obtained from the mushrooms of the *Lentinula edodes* strain 13 presented the highest values of nutritional parameters such as protein and fiber, antioxidant capacity and antimicrobial activity against *Salmonella typhimurium*.

The sensory test, based on a nine-point survey provided to the panelists, showed a general acceptability for the flour sample of mixture 2, composed of 80% mushroom flour of the *Lentinula* strain 13 and 20% CPHF.

The results obtained from this research encourage the production of *Lentinula edodes* using banana leaves as part of the substrate, and promote the use of cocoa pod husks to as an ingredient in the development of new food and seasoning products.

The use of multivariate statistical techniques allows a better visualization of the flour samples with the highest antioxidant capacity and antimicrobial activity for use in the pharmaceutical field.

## Figures and Tables

**Table 1:** Sensory quality evaluation of the mixtures of flours.

Sensory Attribute	Mushrooms Flour (*L. edodes* Strain 13)	CPH Flour	Mixture 1	Mixture 2
**Aroma**	8.5 ± 0.50 ^a^	7.7 ± 0.80 ^b^	8.1 ± 0.75 ^a^	8.3 ± 0.63 ^a^
**Color**	8.7 ± 0.17 ^a^	5.1 ± 0.15 ^c^	6.4 ± 0.83 ^b^	8.5 ± 0.42 ^a^
**Texture**	7.2 ± 0.24 ^b^	2.1 ± 0.42 ^c^	7.2 ± 0.67 ^b^	7.9 ± 1.10 ^a^
**Acceptability**	6.8 ± 0.12 ^b^	2.9 ± 0.17 ^c^	7.0 ± 0.91 ^b^	8.1 ± 1.10 ^a^

Mixture 1 = 60% mushroom flour from *Lentinula edodes* strain 13 mixed with 40% cocoa pod husk flour; mixture 2 = 80% mushroom flour from *Lentinula edodes* strain 13 mixed with 20% cocoa pod husk flour. Different letters in each raw value indicate significant differences between the sensory attributes at level *p* < 0.05, according to Duncan’s test; n = 50 panelists.

## Data Availability

Not applicable.
